# Professional capacity building of Health Science Journal Editors

**DOI:** 10.12669/pjms.35.4.1299

**Published:** 2019

**Authors:** Shaukat Ali Jawaid, Masood Jawaid

**Affiliations:** 1 Shaukat Ali Jawaid Chief Editor, Pakistan Journal of Medical Sciences, Karachi - Pakistan; 2 Masood Jawaid Associate Editor, Pakistan Journal of Medical Sciences, Karachi - Pakistan

A very important development has taken place in the field of Medical Journalism in Pakistan. University of Health Sciences (UHS) Lahore has finally announced to start a Certificate Course in Medical Editing. The curriculum for the course, teaching and training methodology as well as assessment has been finalized and if all goes well the first batch should be enrolled from September 2, 2019. Leadership of Pakistan Association of Medical Editors (PAME) needs to be congratulated for having realized this dream after deliberations spread over many years. Former Vice Chancellor of UHS Prof. Maj. Gen. Muhammad Aslam at present Pro VC at National University of Medical Sciences (NUMS) deserve appreciation for having contributed a great deal towards the realization of this dream when he was the VC UHS while the present Vice Chancellor of UHS Prof. Javed Akram also deserve to be commended for his keen interest and having pushed this project and got it implemented.

Pakistan Association of Medical Editors (PAME) on its part ever since its inception has been busy in organizing seminars, hands on workshops on medical writing for authors and young researchers, peer review workshops for reviewers besides training courses for editors of biomedical journals with its limited resources. Many members of PAME continue to facilitate such academic activities and it has helped to guide the authors, young researchers and faculty members to acquaint themselves with the art of medical writing and principles of scientific publishing.1-4

## Certificate Course in Medical Editing

Now this initiative by the university of Health Sciences (UHS) Lahore will go a long way in improving the professional capacity of those involved in editing and production, publication of health sciencel journals besides providing an opportunity to those who wish to earn this additional qualification. After detailed deliberations among all the stake holders, some of the decisions which have been taken are as under:

1. It was decided that let us start with a basic Certificate Course in Medical Editing with six months duration.
2. This will be followed by an Advance Course in Medical Editing again with six months duration.
3. These will be on line courses based on two contact session of five days each from 9.00 AM to 5.00 PM daily with Forty Credit hours. Hence two contact sessions will have eighty credit hours in all. The candidates will be given assignments during the course which they will maintain in their respective portfolios. These portfolios will be used for final assessment in addition to short questions paper and viva examination. This portfolio must be signed by the Mentor of the candidate.
4. The candidates will get pre-reading material before the contact session and they will come prepared after reading it.
5. There will be in-course assessment which will comprise of 60% marks while 40% marks will at the end of module on final assignment.
6. Each candidate will be allotted a Mentor who will be responsible for guiding them and offer any help, assistance they need during the course.
7. The candidates will be provided a soft copy of Course Resource book which will contain useful informative material which will be helpful to them during their training.
8. All graduates in medical and allied health sciences i.e. MBBS, BDS, BSc in Nursing and Physiotherapists, pharmacy graduates will be eligible for admission.
9. Selection of the candidates will be based on interview after short listing of those who apply. Admission to the advanced course will be on successful completion of the Basic Course.
10. The course fee will be Rs. 40,000/- (Forty thousand) payable in advance at the time of admission.
11. Apart from the Course Faculty, working journalists from general media may also be invited during the contact period to give their input and address the course participants.
12. Few days on the job training for the course participants at the offices of journals recognized for this training course will be an important part of this course.

### Faculty Members

Initially the following faculty members have been identified:

1. Shaukat Ali Jawaid Chief Editor Pakistan Journal of Medical Sciences, Karachi.
2. Fatema Jawad Editor-in-Chief Journal of Pakistan Medical Association, Karachi
3. Jamshed Akhtar Editor, Journal of College of Physicians & Surgeons Pakistan, Karachi
4. Akhtar Sherin, Chief Editor Khyber Medical University Journal, Kohat, Khyber PK
5. Prof. Saira Afzal, Editor Annals of KEMU Lahore.
6. Masood Jawaid Associate Editor Pakistan Journal of Medical Sciences Karachi.
7. Mr. Atif Director Media and Publications at UHS Lahore.

*Note:* More Faculty members will be co-opted as per requirements.

### Topics to be addressed durning course

- Introduction to medical journalism
- Literature search
- Structured abstract, summary for review article
- Different types of medical writing OA, Reviews, Meta analysis, case reports, special communications etc.
- How to do copy editing
- Magazine production
- Online production
- News and feature writing
- Introduction to print and electronic media
- Different business models
- Introduction to different WHO Regions
- ***Peer Review system:*** Types of Peer Review, single, double blind, Open Peer Review System, their advantages and disadvantages.
- English for medical writing
- Introduction to Plagiarism.
- Journalism skills
- Functional English
- Forms and Platforms of writing
- Introduction to Open Journal System
- Scientific misconduct, plagiarism, fabrication, falsification, duplicate ad redundant publications, how to deal with scientific misconduct
- Authorship problems and citation amnesia
- ICMJE authorship guidelines
- Introduction to different bodies of editors i.e. ICMJE, COPE, WAME, EMAME, APAME

## Advance Certificate Course in Medical Editing

*(Only those who qualify the Certificate Course will be eligible to get enrolled in this course)*.

### Topics to be addressed durning course

- Retraction How to Handle it.
- Similarity index, how to interpret it
- How to minimize plagiarism
- Practical application how to use Turnitin, iThenticate
- Advertisement policy.
- Communication with authors
- Rights and Responsibilities of Editors
- Rights and Responsibilities of Publishers
- Editorial Board, National, International Advisory Boards
- Bibliography, Reference management,
- ENDNOTE
- Indexing services, PubMed, PubMed Central, Medline etc.
- Impact Factor, ISI Web of Sciences
- Research Methodology
- Tables, Figures, their Relevance
- Research Writing
- Introduction to various Trial Registries
- Equator statement of RCTs
- Scientometrics IF, Hirsch Index, other indexes
- Legal issues faced by Journals.
- Conflict of Interest in Medical Journalism
- Conflict resolution in Medical Journalism
- Prepare a brief note on how you would like to implement the experience gained to improve the standard of your journal. What will be your short term goals and long term Goals.
- Critical analysis of one of the latest issues of any journal. Highlight the deficiencies and give suggestions to improve it.
- Write a Review on any book published by some local author
- Write your impressions of time spent in a Journal office describing how useful it has been to achieve the objectives of this course.
- Write a feature on any medical institution with particular emphasis on its contributions to promote the art of medical writing and scientific publishing.

Most of the medical universities and medical, dental colleges have now started publishing their own journals while others are planning to do so in the days to come. Hence this course will be extremely helpful in providing them the well trained human resource which is essential if they have to maintain some quality and standard of their journals and also ensure sustainability. For this these institutions must have some minimum dedicated staff.^6^ So far there was no facility for training of these young editors and UHS has fulfilled this vacuum with the start of this course.

Various bodies of editors in different regions have been organizing conferences to help build the professional capacity of editors. Attending these conferences has been a great learning experience 7-8 but unfortunately since most of those appointed as Editors by journals being published by professional organizations or medical institutions are faculty members who are just given this additional assignment without developing proper infrastructure and training, they fail to do the job efficiently with the result that the quality of these journals vary and it needs lot of improvement. Moreover those faculty members who are though keen to be appointed as Editors but are least interested in attending these conferences of editors at their own as it is a bit expensive and this is not their first priority but their own specialty in medicine. The editors need to have at least the basic professional competencies which are considered essential for those running and managing the affairs of biomedical journals. Now those really interested will find this course available within the country quite helpful.^9^
